# Effective type label-based synergistic representation learning for biomedical event trigger detection

**DOI:** 10.1186/s12859-024-05851-1

**Published:** 2024-07-31

**Authors:** Anran Hao, Haohan Yuan, Siu Cheung Hui, Jian Su

**Affiliations:** 1 School of Computer Science and Engineering, Nanyang Technological University, 50 Nanyang Avenue, Singapore, Singapore; 2 Aural & Language Intelligence, Institute for Infocomm Research, Agency for Science, Technology and Research, 1 Fusionopolis Way, Singapore, Singapore

**Keywords:** Biomedical event trigger detection, Representation learning, Type label semantics, Semantic relationships

## Abstract

**Background:**

Detecting event triggers in biomedical texts, which contain domain knowledge and context-dependent terms, is more challenging than in general-domain texts. Most state-of-the-art models rely mainly on external resources such as linguistic tools and knowledge bases to improve system performance. However, they lack effective mechanisms to obtain semantic clues from label specification and sentence context. Given its success in image classification, label representation learning is a promising approach to enhancing biomedical event trigger detection models by leveraging the rich semantics of pre-defined event type labels.

**Results:**

In this paper, we propose the Biomedical Label-based Synergistic representation Learning (BioLSL) model, which effectively utilizes event type labels by learning their correlation with trigger words and enriches the representation contextually. The BioLSL model consists of three modules. Firstly, the Domain-specific Joint Encoding module employs a transformer-based, domain-specific pre-trained architecture to jointly encode input sentences and pre-defined event type labels. Secondly, the Label-based Synergistic Representation Learning module learns the semantic relationships between input texts and event type labels, and generates a Label-Trigger Aware Representation (LTAR) and a Label-Context Aware Representation (LCAR) for enhanced semantic representations. Finally, the Trigger Classification module makes structured predictions, where each label is predicted with respect to its neighbours. We conduct experiments on three benchmark BioNLP datasets, namely MLEE, GE09, and GE11, to evaluate our proposed BioLSL model. Results show that BioLSL has achieved state-of-the-art performance, outperforming the baseline models.

**Conclusions:**

The proposed BioLSL model demonstrates good performance for biomedical event trigger detection without using any external resources. This suggests that label representation learning and context-aware enhancement are promising directions for improving the task. The key enhancement is that BioLSL effectively learns to construct semantic linkages between the event mentions and type labels, which provide the latent information of label-trigger and label-context relationships in biomedical texts. Moreover, additional experiments on BioLSL show that it performs exceptionally well with limited training data under the data-scarce scenarios.

## Background

For the past decades, biomedical information extraction has significantly contributed to our understanding of human health and disease. Recently, with the rapid development of Natural Language Processing (NLP), biomedical event trigger detection, which enables the mining of structured, organized, and valuable information from unstructured biomedical data sources, has attracted significant attention from the research community. A typical biomedical event extraction process consists mainly of two components: trigger detection and argument extraction [1]. Serving as the fundamental step of biomedical event extraction, the task of trigger detection determines the event types and identifies their triggering words from biomedical texts. Biomedical events often happen according to the occurrence of specific biomedical phenomena or molecules. Recognizing these events holds significant potential benefits, especially in disease prevention, health diagnostics, and drug development. As prior research [2, 3] has shown that a significant portion of event extraction errors is attributed to inaccurate trigger detection, it is particularly important to devise an effective event trigger detection method for biomedical event extraction. Biomedical event trigger detection is different from other event detection tasks due to the distinctive characteristics of biomedical texts. These texts are often dense with domain-specific terminology, where the same term may have varying meanings depending on its context [4]. This ambiguity can make event trigger detection more difficult to handle. Moreover, the rapidly evolving nature of biomedical knowledge [5] poses a further challenge in learning the intricate semantics of the pre-defined event types.

Biomedical event trigger detection is commonly treated as a word-level or span-level classification problem [6]. Current state-of-the-art methods can be broadly classified into feature-based methods [7] and representation-based methods [8]. Although these methods have achieved promising performance, they encounter different challenges when applied to biomedical event trigger detection. Feature-based methods often depend on manual feature engineering, which may limit generalizability and adaptability across diverse datasets [9, 10]. On the other hand, representation-based methods may alleviate the need for creating semantic features manually. With the richness of semantic information inherent in the data, representation-based methods are more versatile and adaptable to different datasets [11–13]. However, such methods [14, 15] often treat event type classes as homogeneous one-hot vectors without considering the rich semantics of event type labels, neglecting their potential correlation relationships with input texts. As such, it may affect the representation learning process, thereby degrading the performance. Furthermore, most current representation-based methods [8, 16, 17] rely heavily on utilizing syntactic parsing tools for enhanced semantic representations. While this approach can improve performance, it also increases time complexity and may potentially lead to over-dependency on external tools or resources.

**Fig. 1:**
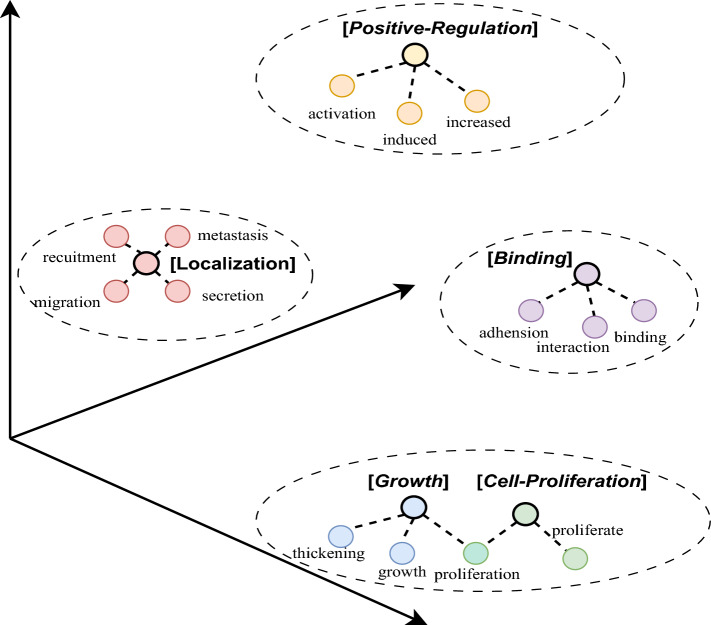
The event type labels (enclosed by square brackets ${\varvec{[\cdot ]}}$) and their trigger words have close proximity in the semantic space, according to the co-occurrence of event types and trigger words in the MLEE dataset

Recent studies on label representation learning, which incorporates event type label words as model input, have shown promising performance in text classification [18, 19] and event detection tasks [20]. However, its application to biomedical event trigger detection has not been fully explored. In biomedical event trigger detection, events are categorized into event types with labels such as Positive-Regulation, Growth and Localization according to the nature of the biomedical events they represent. We have conducted an experiment and performed a statistical analysis to study the semantic association between event type labels and their trigger words based on the training set of the MLEE dataset [3]. We found that event types and their trigger words have close semantic affinity in semantic space. Figure 1 shows an example on the semantic space of type label words and their respective trigger words. For example, the event type Positive-Regulation has close semantic proximity to the trigger words such as “*induced*”, “*increased*”, and “*activation*”. Similarly, event types Growth, Localization, Binding, and Cell-Proliferation also show similar semantic affinity with their trigger words. However, although the word “*proliferation*” is a trigger word for Cell-Proliferation, it is also a trigger word for another type of event, Growth. Therefore, extracting the latent relationships between type labels and trigger words is important for biomedical event trigger detection.

**Fig. 2:**
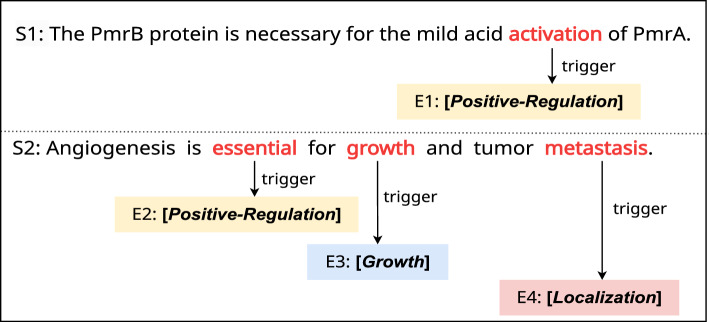
Two example sentences taken from the MLEE dataset. The event types are enclosed by square brackets ${\varvec{[\cdot ]}}$, and the trigger words are highlighted in red

Let’s consider the two sentences S1 and S2 given in Fig. 2, which shows the event types and their corresponding trigger words. In S1, event E1 is identified with event type Positive-Regulation and trigger word *“activation”*. S2 is identified with three events: event E2 with event type Positive-Regulation and trigger word *“essential”*, event E3 with event type Growth and trigger word *“growth”*, and event E4 with event type Localization and trigger word *“metastasis”*. From Fig. 2, we find that the trigger word *“activation”* is commonly associated with the event type Positive-Regulation for event E1. Similarly, the same applies to *“growth”* and Growth for event E3, and *“metastasis”* and Localization for event E4. However, the event type Positive-Regulation and the trigger word *“essential”* do not have a close semantic relationship for event E2. Instead, the contextual relationships between event type Positive-Regulation and other words such as *“angiogenesis”* and *“growth”* in S2 provide important clues for event trigger detection. Therefore, apart from the type label and trigger word relationships, the relationships between the type label and the contextual words in the sentence also provide important information for biomedical event trigger detection.

In this paper, we propose an effective model called Biomedical Label-based Synergistic representation Learning (BioLSL), which learns label-trigger and label-context relationships, for biomedical event trigger detection. The proposed BioLSL model comprises three modules, namely Domain-specific Joint Encoding, Label-based Synergistic Representation Learning, and Trigger Classification. First, the Domain-specific Joint Encoding module uses the biomedical domain-specific pre-trained PubMedBERT [21] model to jointly encode the input sentence and type labels into representations through the self-attention mechanism. Next, the Label-based Synergistic Representation Learning module formulates the type label and input sentence representations by using an interaction matrix and attention mechanism. In particular, we extract the Label-Trigger Aware Representation (LTAR) to capture the semantic relationships between the event types and their corresponding potential triggers, and the Label-Context Aware Representation (LCAR) to capture the relationships between the type labels and the contextual words in biomedical texts. Then, both semantic representations are mapped into a consistent dimensional space for extracting the latent label-trigger and label-context relationships for biomedical event trigger detection. Finally, the Trigger Classification module uses the Conditional Random Field (CRF) [22] to decode the combined semantic representation for predicting the event triggers.

Overall, the main contributions of the paper are summarized as follows: (1) We propose an effective label representation learning model called BioLSL for biomedical event trigger detection. Different from the current state-of-the-art models, BioLSL utilizes pre-defined event type labels to learn the semantics from intricate biomedical texts without relying on any external resources. (2) We design an effective method to synergistically learn the label-trigger and label-context relationships to enhance the process of biomedical event trigger detection. (3) We evaluate the performance of the proposed BioLSL model on three widely used benchmark datasets, namely MLEE, GE09 and GE11. The performance results show that the proposed BioLSL model has achieved state-of-the-art performance, outperforming the existing baseline models with an improvement of 1.04–2.78% in absolute F1-scores. Moreover, we also demonstrate that the BioLSL model is able to achieve competitive performance with limited training data under data-scarce scenarios.

The rest of the paper is organized as follows. Section 2 reviews the related work on biomedical event trigger detection and label representation learning. Section 3 presents the details of the proposed BioLSL model for biomedical event trigger detection. Section 4 discusses the performance results. Finally, Sect. 5 concludes the paper.

## Related work

Biomedical event trigger detection has been investigated over the past decade. However, most of the previous methods [16, 23–25] rely heavily on external syntactic parsing tools without considering the semantics of pre-defined type labels. Recently, label representation learning [18, 19, 26, 27] has received much attention in the research community. In this section, we review the related work on biomedical event trigger detection and label representation learning.

### Biomedical event trigger detection

The earlier works on biomedical event trigger detection mainly focus on feature-based techniques [28, 29], converting classification cues into feature vectors through a variety of strategies [30]. HASH [31] proposed using hash operations to convert dependency graph structures into features for enhancing event trigger detection. SVM-CRF [9] integrated the classification capabilities of Support Vector Machine (SVM) with the sequence handling capabilities of Conditional Random Field (CRF), which allows the model to detect event triggers in sequential data effectively. Bio-SVM [10] designed a feature engineering process to extract syntactic and semantic contextual features, and combined them with domain-specific knowledge to enhance the detection of event triggers. TSVM [7] implemented a two-stage SVM classifier that incorporates feature selection with word embeddings to provide a syntactically rich representation of words, thereby enhancing the performance for biomedical event trigger detection. However, these methods mainly utilize manual features, which can only achieve limited generalizability.

In recent years, progress in representation learning and neural networks has led researchers to integrate language representation learning (e.g., Glove [32] and BERT [33]) and domain-specific learning [34] into neural models for event trigger detection. For example, BiLSTM-FastText [35] incorporated FastText embeddings into a bidirectional long short-term memory model (BiLSTM), which allows the model to extract unsupervised features and identify sequence relationships among words. To fully utilize the contextual and temporal information, AttBiLSTM [36] implemented BiLSTMs with the attention mechanism to capture contextual semantics of words and entity types for biomedical event detection. To further exploit the potential of the attention mechanism for biomedical trigger detection, AttGRU [37] proposed a gated mechanism and an attention-based GRU encoder for contextual semantic representation. DeepEventMine [11] proposed an end-to-end framework based on BERT-based contextual word embeddings and named entity information to jointly extract multiple biomedical events including event triggers and arguments. BioKGLM [38] integrated the structural knowledge graph into contextualized BERT-based models to improve the performance of the biomedical information extraction task. ResLSTM [23] deployed a gated multi-layer residual BiLSTM with a CRF layer to dynamically compute contextualized word representation while preserving sequence dependencies for event trigger identification. These works focus mainly on utilizing semantic and contextual information for biomedical event trigger detection.

Recently, some works have started exploring the use of dependency information in neural network models to improve the performance of biomedical event trigger detection. Inspired by [39], TEES-CNN [25] enhanced the Turku Event Extraction System that integrates multiple CNNs to capture the local dependencies in text, and employs several pre-trained embeddings for different feature extractions for biomedical event trigger detection. Similarly, RecurCRFs [16] combined a dependency-tree-based RNN with a CRF layer to model sentence semantics for event trigger detection. To further investigate the importance of dependency information, Fei et al. [40] proposed to use graph neural network for recognizing the relationships between biomedical entities for better representation learning in biomedical event trigger detection. Moreover, Tree-LSTM [8] is the state-of-the-art BERT-based model, which employs BioBERT [12] as the encoder and a LSTM layer that uses dependency tree features for deep and context-aware understanding of biomedical semantics for biomedical event trigger detection. While these methods have achieved competitive performance, they still require additional linguistic tools to obtain dependency embeddings for constructing syntactic features [41, 42], which may potentially introduce noise into the model. Furthermore, the syntactic parsing methods may not be applicable across different biomedical event datasets.

### Label representation learning

Label representation learning has been a prevalent approach for image classification, but its application for natural language processing (NLP) tasks remains relatively underexplored. For a few studies in [18, 19, 26] that have ventured into this area, label information has been encoded as system input for text classification. Moreover, Ngo et al. [43] also proposed to encode the relation and connective labels for discourse relation recognition. However, many of these methods have limitations, as they rely on separate encoders for labels and input sentence words. This can lead to redundancy, as both labels and sentences are originated from the same English vocabulary and could potentially share the same embeddings. Furthermore, existing methods are often unable to effectively capture the intricate interactions between sentence words and event type labels. To address these issues, the proposed BioLSL model adopts a unified encoding scheme for both input texts and label words. It also utilizes self-attention mechanisms to capture the high-level interactions between these words.

Although some previous works, such as those by Zhang et al. [27] and Huang et al. [44], use pre-trained language models for label representation learning, these works do not directly employ type label words to enhance the representation of the relationships between input texts and type labels. For instance, Zhang et al. [27] tackled zero-shot event extraction by using label words as seeds to manually curate “example trigger words” from a large external corpus, and meanwhile Huang et al. [44] proposed to learn latent type representations from input sentences for both supervised and semi-supervised event detection. Despite these efforts, both works have not established a robust semantic linkage between type labels and input word representations. To utilize semantic information from event type labels, SemPRE [20] proposed a supervised contrastive learning framework to learn the contrastive relationships between trigger words and type labels. However, defining negative samples to learn contrastive information [45] based on biomedical event datasets poses another challenge for biomedical event trigger detection. Apart from that, most of these previous works have not fully considered the interactive attention [46] between the input texts and type labels and the context-dependent semantics of type labels.

### Discussion

In summary, traditional feature-based methods have limited generalizability, while neural network-based methods leverage representation learning to improve performance but often do not integrate dependency information. Recent methods that incorporate dependency information lead to performance improvement to a certain degree, but these methods introduce noise and face dataset generalizability issues. In contrast, we devise an effective yet efficient framework to explicitly enhance semantic representation using pre-defined event type labels, which achieves better performance.

For label representation learning methods, separate encoding schemes may lead to redundancy and limited interaction, while unified encoding schemes like our proposed BioLSL model address these issues by capturing high-level interactions and enhancing semantic representation for biomedical event trigger detection. Most existing works that explore pre-trained language models for label representation learning are unable to establish a robust semantic linkage between type labels and input word representations. There have been efforts to address this issue using contrastive learning, but for biomedical event detection such an approach faces challenges such as defining negative samples. To address these gaps, our proposed BioLSL model introduces the Label-Trigger Aware Representation and the Label-Context Aware Representation for effective semantic representation for biomedical event trigger detection.

## Methods

**Fig. 3:**
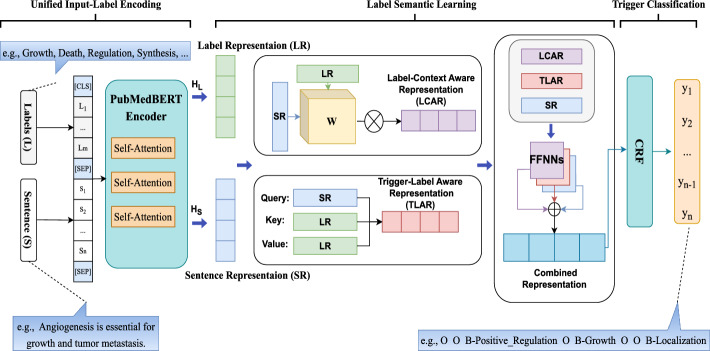
Architecture of our proposed BioLSL model

Figure 3 shows the overall architecture of the proposed Biomedical Label-based Synergistic representation Learning (BioLSL) model for biomedical event trigger detection. The proposed BioLSL model takes in a sentence *S* in the form of $\{s_1, s_2,..., s_n\}$, where $s_i$ is the *i*-th token in the sentence and *n* denotes the total number of tokens, and $T_{BED} = \{t_1, t_2,..., t_k\}$ is a set of pre-defined event type labels, where *k* denotes the total number of event types. The model outputs a predicted label sequence *L* in the form of $\{l_1, l_2,..., l_n\}$, where $l_i$ denotes the type label for $s_i$, and $l_i \in T_{BED}$. Moreover, we use the BIO tagging scheme [47] in our model to mark the trigger words consisting of multiple tokens. The BIO tagging scheme is a tagging format for representing labels in a sequence, which uses the following three tags: “*B*” (beginning of the event trigger), “*I*” (inside of the event trigger) and “*O*” (outside the event trigger). As such, it can maintain the boundaries between adjacent event triggers. For example, given the sentence “*Angiogenesis is essential for growth and tumor metastasis.*”, the model produces the output * {O, O, B-Positive_Regulation, O, B-Growth, O, O, B-Localization}* according to the BIO tagging scheme.

The proposed BioLSL model consists of three modules, namely Domain-specific Joint Encoding, Label-based Synergistic Representation Learning and Trigger Classification.

### Domain-specific joint encoding

This module takes in the input sentences and a list of pre-defined type labels for encoding. In particular, the biomedical pre-trained language model PubMedBERT [21] is used to encode each word in the input sentence, which is represented as:

1 $$\begin{aligned} X_{L,S} = \langle [CLS],L,[SEP_1],S,[SEP_2] \rangle \end{aligned}$$

where *L* is the type label words, *S* is the input sentence, and [CLS] and [SEP] are special tokens in PubMedBERT. Note that *L* is a fixed list of text names describing the pre-defined event types’ concepts. For example, the MLEE [3] biomedical corpus contains 19 pre-defined event types such as Growth, Death, Regulation, etc. and *L* is created as a random sequence of type labels, e.g., {Growth, Death, Regulation, Synthesis,... } . For the few type labels whose names contain multiple words, we employ additional special tokens to represent them.

Inspired by [20], the Domain-specific Joint Encoding module uses PubMedBERT’s multi-head self-attention mechanism [48] to capture the direct interactions between the type labels *L* and the input sentence *S*. Then, the representation for the type labels and the sequential representation for input sentence tokens are generated through the multiple Transformer layers. The attention heads in the Transformer layers can be expressed as follows:

2 $$\begin{aligned} ATTENTION(Q,K,V)=softmax\left( \frac{QK^T}{\sqrt{d_k}}\right) V \end{aligned}$$

where $d_k$ denotes the encoding dimension, and *Q*, *K*, *V* represent the query, key, and value matrices, respectively.

Then, PubMedBERT is used to generate the representations for the type labels and the input sentence:

3 $$\begin{aligned} (H_L,H_S) = PubMedBERT(X_{L,S}) \end{aligned}$$

where $H_L$ is the encoded token sequence of type label words, and $H_S$ is the encoded token sequence of the input sentence, called Label Representation (LR) and Sentence Representation (SR), respectively. These two representations are derived by splitting the output representation from PubMedBERT.

### Label-based synergistic representation learning

In biomedical event trigger detection, predicting event triggers is highly context-dependent. For example, as pointed out by Pyysalo et al. [3], a term is considered indicative of a Growth event only when it can distinctively make reference to the “upper-level gene ontology”. To capture such contextual information, it is essential to capture the meaningful contextual input sentence tokens and match them with their corresponding type label tokens. Therefore, we first obtain the Label-Context Aware Representation (LCAR), *C*, that captures the intricate interaction of the contextual input sentence tokens with the event types.

The label-context aware representation $C_{i}$ of input sentence token $S_i$ is computed as follows:

4 $$\begin{aligned} \begin{aligned} C_{i} = \frac{1}{N} \sum _{j=1}^N {\tilde{a}}_{ij} \cdot h_L^{(j)} \\ {\tilde{a}}_{ij} = \theta (h_S^{(i)},h_L^{(j)}) \end{aligned} \end{aligned}$$

where *N* is the total number of type labels, and $\theta (.)$ denotes the attention function, which is computed using the input sentence representation for query, and the type label representation for both key and value.

After that, to determine whether an input sentence token $h_S^{(i)}$ is the candidate trigger of a certain event type, we need to calculate the semantic proximity and capture the underlying semantic relationship between the latent trigger tokens in input sentence and the target type label tokens. Therefore, we compute an interaction matrix, *W*, which links the encoded token sequence of type label words (i.e., $H_L \in {\mathbb {R}}^{m \times d_k}$), with an encoded token sequence of the input sentence (i.e., $H_S \in {\mathbb {R}}^{n \times d_k}$). Here, *m* represents the total number of type label words, *n* refers to the number of tokens in the input sentence, and $d_k$ indicates the encoding dimension. Then, it generates the Label-Trigger Aware Representation (LTAR), $A_{i,j}$, for each pair of a type label and an input sentence token $<S_i,L_j>$ as follows:

5 $$\begin{aligned} A_{i,j} = \sigma ({h_S^{(i)}}^\top W h_L^{(j)} + b) \end{aligned}$$

where $\sigma$ denotes the sigmoid nonlinearity function and *b* is a bias term. The interaction matrix *W* is learnable and continues to update during training. Thus, the matrix can capture the underlying semantic relationships between the type labels and their corresponding trigger words.

Besides the original input sentence representation (i.e., $H_S$), our proposed model also contains the semantic information from the label-context aware representation and label-trigger aware representation. These two representations learn latent information from label-trigger and label-context relationships to help the detection of biomedical event triggers. We map these three representations (i.e., $H_S$, *C* and *A*) to a Conditional Random Field (CRF) [22] for decoding by using three separate single layer feed-forward neural networks (FFNNs) with cross-type activation functions:

6 $$\begin{aligned} \begin{aligned} X'&= FFNN_{1}(H_S) \\ {\hat{X}}&= FFNN_{2}(C)\\ {\tilde{X}}&= FFNN_{3}(A) \end{aligned} \end{aligned}$$

where $X'$, ${\hat{X}}$, and ${\tilde{X}}$ represent the corresponding mapped representations, with each being a matrix in ${\mathbb {R}}^{n \times k}$, where *k* refers to the number of type labels based on the BIO tagging scheme. Most previous works [49] concatenate different representations for joint decoding. However, it may potentially lead to overly sparse feature representations and cause the problem on gradient vanishing. As concatenation increases the dimensionality of the feature space, it will render the features more sparsely in the high-dimensional space [50]. The sparsity poses a significant challenge to the model’s learning capability, as the model will struggle to learn patterns from the sparse features and generalize them accordingly. This is particularly problematic in biomedical event trigger detection, where the number of training samples is relatively small compared to the vast feature space [51]. Therefore, we use different FFNNs to map each representation individually.

We combine the separate representations by applying a weight parameter to balance each representation’s contribution. Drawing inspiration from the work on residual learning [52], we aim to ensure the preservation of valuable information and avoid potential model degradation. In particular, as discussed in [53, 54], the sentence representation output from BERT carries important contextual information. Therefore, we balance and combine the different representations, denoted as $x = \{x_1,x_2,...,x_n\}$, as follows:

7 $$\begin{aligned} x_i = x'_i + \alpha {\hat{x}}_i + (1-\alpha ) {\tilde{x}}_i \end{aligned}$$

where $x'_i \in X'$, ${\hat{x}}_i \in {\hat{X}}$, and ${\tilde{x}}_i \in {\tilde{X}}$. And $\alpha \in (0,1)$ is a hyperparameter to be determined empirically.

### Trigger classification

This module uses CRF for identifying event trigger candidates by decoding the combined representation and predicting the event trigger. Since our proposed model makes use of the BIO tagging scheme for modeling the type labels, it is important to consider label sequence. For example, the label “I” should not directly follow the label “O”. Activation functions such as Softmax are unable to take label dependencies and label sequence into consideration for prediction.

Given the combined representation *x* obtained from the Label-based Synergistic Representation Learning module, CRF computes the probability of a ground truth type label sequence $y= \{y_1,y_2,...,y_n\}$ as follows:

8 $$\begin{aligned} P(y\mid x)= & {} \frac{exp \big ( score(x,y) \big )}{\sum _{y' \in Y} exp \big ( score(x,y') \big )} \end{aligned}$$

9 $$\begin{aligned} score(x,y)= & {} \sum _{i=0}^{n-1} T_{y_i, y_{i+1}} + \sum _{i=0}^{n} F_{x,y_i} \end{aligned}$$

where *n* is the length of *x*, *Y* is a set of all possible type label sequences, and $y'$ is the predicted type label sequence. *T* is the transition matrix, with $T_{y_i,y_{i+1}}$ being the transition parameter from label $y_i$ at position *i* to label $y_{i+1}$ at position $i+1$. $F_x$ is the emission matrix of the representation *x*, with $F_{x,y_i}$ being the score of label $y_i$ at position *i* with respect to *x*.

After that, we use the Negative Log-Likelihood loss function [55] to measure the distance between the predicted type label sequence and the true type label sequence:

10 $$\begin{aligned} {\mathcal {L}}(x, y) = -\log P(y|x) \end{aligned}$$

where *y* is the ground true type label sequence and *x* is the combined representation. Finally, our proposed BioLSL model minimizes the loss function ${\mathcal {L}}(x, y)$ during training by optimizing the parameters of the proposed model.

For prediction, we apply the argmax function to the probability distribution $P(Y\mid X_{L,S})$ to obtain the predicted type label sequence $y^*$ as follows:

11 $$\begin{aligned} y^* = \mathop {argmax}P(Y \mid X_{L,S}) \end{aligned}$$

## Results

In this section, we first discuss the datasets, baseline models, implementation details and evaluation measures. Then, we present the experimental results of the proposed model and the baseline models for the biomedical event trigger detection task, and analyze the results in details.

### Datasets

We conduct the experiments based on the following three benchmark datasets for the biological trigger detection task:

- MLEE [3]—It is derived from a collection of 262 PubMed abstracts. MLEE is the most widely used benchmark dataset for the biological trigger detection. This dataset includes a diverse range of biomedical events covering all levels of biomedical organizations from the molecular to the organismal levels. It consists of 19 pre-defined event types. We use the train/dev/test split given by the data provider.
- GE09 [56]—It is obtained from the BioNLP-09 Shared Task, focusing on the identification of molecular events present in biomedical literature. The dataset is based on the extensively annotated GENIA corpus. It contains 9 pre-defined event types. We use the train/dev/test split given by the shared task, and evaluate the performance based on the development set, as the test set is unannotated and the official tool for evaluation is no longer available.
- GE11 [57]—It is sourced from the BioNLP-11 Shared Task. The GE11 dataset focuses on events that are related to the transcription factors in human blood cells domain [2]. Like its predecessor GE09, GE11 is based on the extensively annotated GENIA corpus and retains the same 9 pre-defined event types, but it deals with the different articles that are not included in GE09. Similar to GE09, we use the train/dev/test split defined by the shared task, and evaluate the performance based on the development set.

The details of the datasets and splits are summarized in Table 1. For the evaluation metrics, we adopt precision, recall and F1-score. Following the previous work [16], we report the micro-average scores for MLEE and the macro-average scores for GE09 and GE11.

**Table 1:** Statistics of the datasets

	MLEE			GE09			GE11		
	Train	Dev	Test	Train	Dev	Test	Train	Dev	Test
# Documents	131	44	87	800	150	260	908	259	347
# Sentences	1527	438	1027	7449	1450	2447	8691	2900	3371
# Events	3121	670	1894	8597	1809	–	10310	3250	–

###  Implementation details

We implement our proposed BioLSL model using Pytorch [58]. Specifically, we use the base uncased version of PubMedBERT trained on abstracts and full-text articles. The hyperparameter $\alpha$ is tuned during the development process, with the final setting of $\alpha = 0.5$. We fix the maximum sequence length for the datasets to 256 and limit the training to 100 epochs with a learning rate of 5e−5. Both the attention and dense layers utilize the Adam optimizer [59] with a dropout of 0.9 and are updated during training. All of our experiments are conducted on the same machine with Intel(R) Core(TM) i7 CPU@2.10 GHz and a single Nvidia GeForce-RTX 3080Ti GPU.

### Baseline models

The baseline models include Large Language Models (LLMs), feature-based learning models, and representation-based learning models. Large Language Models such as ChatGPT^1^ have been applied for various NLP tasks under zero-shot and few-shot scenarios [60, 61]. Following [62], we also design zero-shot prompts and few-shot in-context learning (ICL) [63] prompts, and use the OpenAI API from ChatGPT-3.5 and ChatGPT-4 [64] for biomedical event trigger detection for performance comparison. In addition, the feature-based learning models including HASH [31], SVM-CRF [9], Bio-SVM [10], and TSVM [7], and the representation-based learning models including BiLSTM-FastText [35], DeepEventMine [11], TEES-CNN [25], RecurCRFs [16], SemPRE [20], ResLSTM [23] and Tree-LSTM [8] are also used as the baseline models. The reported results in Table 2 are obtained from the respective reference papers, except LLMs and SemPRE, which are reconstructed for performance evaluation.

### Experimental results

**Table 2:** Experimental results based on the MLEE, GE09 and GE11 datasets

Methods	MLEE			GE09			GE11		
	P $(\%)$	R $(\%)$	F1 $(\%)$	P $(\%)$	R $(\%)$	F1 $(\%)$	P $(\%)$	R $(\%)$	F1 $(\%)$
*Large language models (LLMs)*									
ChatGPT-3.5 (0-shot)	33.02	30.17	31.53	17.53	26.51	21.10	14.69	28.00	19.27
ChatGPT-4 (0-shot)	35.40	34.48	34.93	17.92	27.01	21.55	15.28	29.33	20.09
ChatGPT-3.5 (5-shot ICL)	43.75	40.24	41.92	20.54	29.50	24.22	23.53	32.00	27.12
ChatGPT-4 (5-shot ICL)	44.63	42.10	43.33	21.46	31.07	25.39	24.51	33.33	28.25
*Feature-based supervised learning models*									
HASH [31]	–	–	–	**79**.**83**	56.02	65.84	–	–	–
SVM-CRF [9]	–	–	–	69.96	64.28	67.00	–	–	–
Bio-SVM$\dagger$ [10]	75.56	81.29	78.32	–	–	–	–	–	–
TSVM$\dagger$ [7]	80.35	79.16	79.75	75.94	68.31	71.01	68.09	**76**.**41**	72.01
*Representation-based supervised learning models*									
BiLSTM-FastText [35]	77.89	78.28	78.08	68.21	58.55	63.01	68.44	65.26	66.81
DeepEventMine [11]	79.37	78.86	79.12	–	–	–	72.05	68.89	70.43
TEES-CNN [25]	81.49	78.43	79.93	–	–	–	73.32	68.72	70.95
RecurCRFs [16]	81.12	79.15	80.28	76.42	70.45	73.24	–	–	–
SemPRE [20]	79.73	81.44	80.58	71.70	71.99	71.42	73.36	70.83	71.93
ResLSTM [23]	79.89	81.61	80.74	–	–	–	–	–	–
Tree-LSTM [8]	**82**.**24**	80.20	81.21	–	–	–	–	–	–
BioLSL (Ours)	80.71	**83**.**79**	**82**.**25**	74.51	**76**.**34**	**75**.**41**	**78**.**37**	71.67	**74**.**79**

The best results are highlighted in bold

Table 2 shows the experimental results of the proposed BioLSL and the baseline models based on the MLEE, GE09 and GE11 datasets. As shown in Table 2, the BioLSL model has achieved 82.25%, 75.41% and 74.79% in F1-score on the MLEE, GE09 and GE11 datasets, respectively. It outperforms all the baseline models in terms of F1-score on the three datasets. As can be seen from Table 2, the LLMs (e.g., ChatGPT-3.5 and ChatGPT-4) perform worse than the supervised baseline models. With few-shot ICL prompts, ChatGPT is able to improve the performance significantly for biomedical event trigger detection. Although ChatGPT (powered by GPT-3.5 and GPT-4) shows promising performance for certain NLP tasks such as machine translation [65] and text summarization [66], its performance for the biomedical event trigger detection task is still lagging behind the supervised baseline models.

Among the supervised learning models, we can observe that representation-based methods generally achieve better performance than feature-based methods. For the feature-based methods, TSVM has achieved the best performance for the three datasets. This may due to its extensive feature extraction process and the utilization of the Turku Event Extraction System (TEES). Compared with TSVM, the BioLSL model has achieved better F1-score performance on the three datasets. More specifically, BioLSL demonstrates an improvement of 3.5%, 4.40% and 2.78% in F1-score over TSVM on the MLEE, GE09 and GE11 datasets, respectively. Among the representation-based methods, Tree-LSTM, RecurCRFs and TEES-CNN have achieved the best performance on the MLEE, GE09 and GE11 datasets, respectively. However, even though BioLSL does not use any dependency parsing tools as Tree-LSTM, RecurCRFs and TEES-CNN, it still outperforms Tree-LSTM, RecurCRFs, and TEES-CNN by 1.04%, 2.17%, and 3.84% in F1-score on the MLEE, GE09, and GE11 datasets, respectively.

Overall, BioLSL has achieved promising performance on the MLEE, GE09 and GE11 benchmark datasets. The outstanding performance of BioLSL can be attributed to its effective use of type label semantics, which can improve the performance quite effectively for biomedical event trigger detection.

### Ablation study

**Table 3:** Ablation study based on the MLEE, GE09 and GE11 datasets

Model	MLEE		GE09		GE11		Average
	F1$(\%)$	$\triangle$ F1$(\%)$	F1$(\%)$	$\triangle$ F1$(\%)$	F1$(\%)$	$\triangle$ F1$(\%)$	$\triangle$ F1$(\%)$
BioLSL	82.25	–	75.41	–	74.79	–	–
w/o LSRL	81.38	− 0.87	74.24	− 1.17	73.65	− 1.14	− 1.06
w/o DJE+LSRL	80.90	− 1.35	73.65	− 1.76	72.96	− 1.83	− 1.65
w/o LCAR	81.87	− 0.38	74.88	− 0.53	74.17	− 0.62	− 0.51
w/o LTAR	81.73	− 0.52	74.73	− 0.68	74.34	− 0.45	− 0.55

We conduct an ablation study of the proposed BioLSL model to evaluate the effect of its different components on the overall performance based on the MLEE, GE09, and GE11 benchmark datasets. Table 3 shows the performance results of the ablation study. The performance results are reported in terms of F1-score. $\triangle$ F1 indicates the difference in F1-score between the different configuration models and the proposed BioLSL model. As shown in Table 3, the removal of the Label-based Synergistic Representation Learning module (i.e., w/o LSRL) from BioLSL results in a reduction of 1.06% in the average F1-score, highlighting the important role of this module to the model’s overall performance. When both the Domain-specific Joint Encoding (DJE) and Label-based Synergistic Representation Learning (LSRL) modules are removed (i.e., w/o DJE+LSRL) from BioLSL, the average F1-score is then decreased by 1.65%. Therefore, these two modules are important for biomedical event trigger detection. We further study the importance of the two semantic representations embedded by the Label-based Synergistic Representation Learning module, namely Label-Context Aware Representation (LCAR) and Label-Trigger Aware Representation (LTAR). The removal of LCAR (i.e., w/o LCAR) leads to a decrease of 0.51% in the average F1-score. Similarly, removing LTAR (i.e., w/o LTAR) results in a decrease of 0.55% in the average F1-score. The drop in performance indicates that these two kinds of semantic representations can contribute to the performance improvement of the proposed BioLSL model. Overall, each component of the BioLSL model plays an important role in achieving promising performance for biomedical event trigger detection.

### Performance analysis based on various pre-trained models

We evaluate the performance of the biomedical domain-specific pre-trained BERT models as the encoder in the proposed BioLSL model for biomedical event trigger detection. The pre-trained BERT models include the cased and uncased versions of SciBERT [67], BioBERT [12], and PubMedBERT [21]:

- SciBERT—It is a BERT model pre-trained on a dataset of 1.14 million of scientific full-text articles gathered from Semantic Scholar.
- BioBERT—It is a specialized biomedical language representation model, which was developed by the DMIS Laboratory at Korea University for biomedical text mining.
- PubMedBERT—It is a dedicated biomedical language model that was pre-trained on PubMed abstracts [68] and PubMed Central full-text articles [69].

**Table 4:** Performance results of the BioLSL model with various pre-trained models based on the MLEE dataset

Model	P$(\%)$	R$(\%)$	F1$(\%)$
BioLSL$_{BERT-base(uncased)}$	79.44	82.68	81.03
BioLSL$_{SciBERT(cased)}$	80.13	82.83	81.46
BioLSL$_{BioBERT}$	80.14	83.36	81.72
BioLSL$_{BERT-large(uncased)}$	80.09	83.46	81.77
BioLSL$_{SciBERT(uncased)}$	80.21	83.72	81.92
BioLSL$_{PubMedBERT}$	**80**.**71**	**83**.**79**	**82**.**25**

The best results are highlighted in bold

Table 4 shows the performance results of the BioLSL model with various pre-trained models based on the MLEE dataset. All the domain-specific pre-trained models, including SciBERT (cased), BioBERT, SciBERT (uncased) and PubMedBERT, outperform the pre-trained BERT (uncased) model. In particular, the BioLSL model with PubMedBERT has achieved the best performance with 82.25% in F1-score. It outperforms BioLSL with the pre-trained SciBERT (uncased) by 0.33%, BioBERT by 0.53% and SciBERT (cased) by 0.79% in F1-score. Additionally, the BioLSL model with BERT-large (uncased) has gained an improvement of 0.74% in F1-score compared to the BioLSL model with BERT-base (uncased). Overall, the proposed BioLSL model has achieved the best performance when using the pre-trained PubMedBERT model as the encoder.

**Fig. 4:**
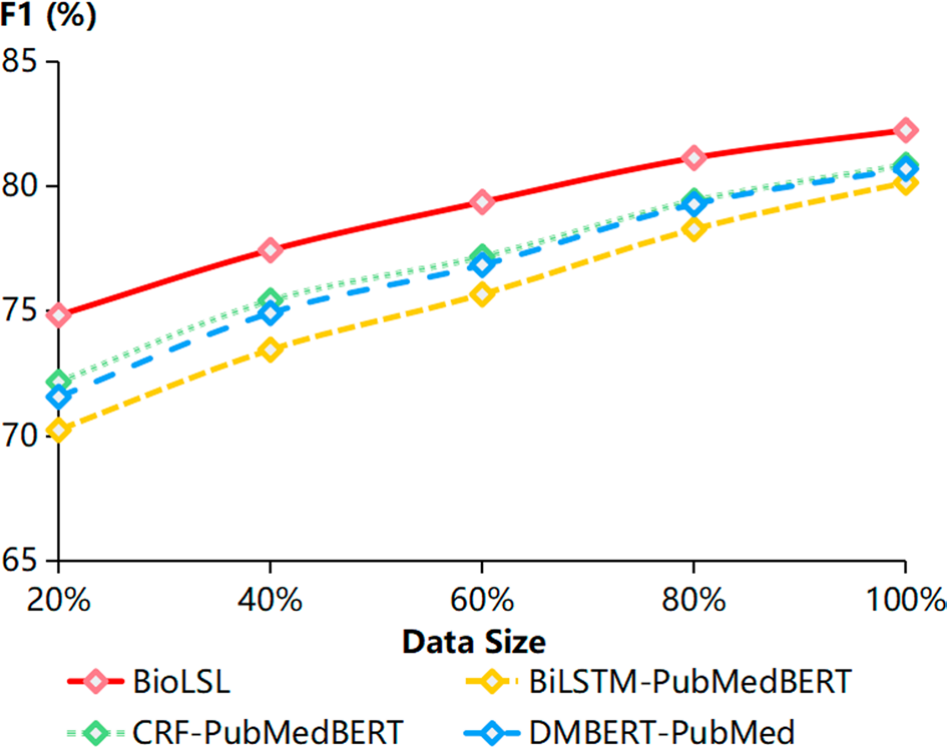
Performance results according to various training data sizes

### Performance analysis based on various data sizes

We analyze the performance of the proposed BioLSL model in comparison with the baseline models in the challenging scenario of data scarcity. The baseline models include BiLSTM-PubMedBERT, CRF-PubMedBERT and DMBERT-PubMed that employ the same pre-trained model, PubMedBERT, as in the proposed BioLSL model. These baseline models are selected for comparison as BiLSTM and CRF are commonly used as the base mechanism for biomedical event trigger detection [16, 24]. In addition, DMBERT [70] is a widely recognized model that adopts a dynamic multi-pooling mechanism for event trigger detection. Although there exist other state-of-the-art biomedical event trigger detection models such as ResLSTM [23] and Tree-LSTM [8], we are unable to use them as baseline models due to the unavailability of their source codes.

In the experiments, we randomly include 20%, 40%, 60%, and 80% of the samples from the training data of the MLEE dataset for performance evaluation. As shown in Fig. 4, the proposed BioLSL model has achieved better performance than the baseline models when the training data size varies from 20% to 100%. In particular, we can observe that with 80% of the training data, BioLSL is able to outperform the baseline models trained with 100% training data. Moreover, when using 60% of the training data, the BioLSL can achieve an F1-score of 79.37%, which is still able to perform quite competitively with other baseline models trained with 100% training data. As can be seen from the performance results, the proposed BioLSL model is able to perform effectively even under the data-scarce scenarios.

**Fig. 5:**
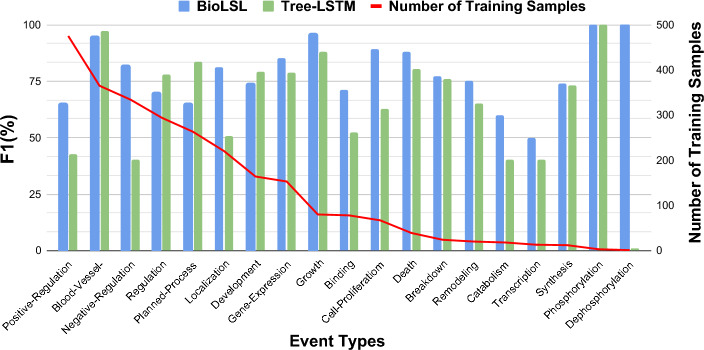
Performance results of BioLSL and Tree-LSTM according to different event types based on the MLEE dataset. The reported results of Tree-LSTM are obtained from [8]

### Performance analysis based on different event types

As discussed in [8], the lack of training samples for some event types could lead to performance degradation due to the difficulty of learning semantic features from such event types for biomedical event trigger detection. Figure 5 shows the performance results of BioLSL and Tree-LSTM on the 19 event types based on the MLEE dataset. From Fig. 5, we can observe that BioLSL outperforms Tree-LSTM on 14 event types, especially those with scarce training samples. For example, BioLSL outperforms Tree-LSTM by 11% and 10% in F1 for the Catabolism and Transcription events, respectively. The Catabolism and Transcription event types contain only 18 and 13 training samples, respectively. This further shows that the proposed BioLSL model is able to perform effectively when only scarce data samples are available for training.

### Performance analysis on the hyperparameter $\alpha$

**Fig. 6:**
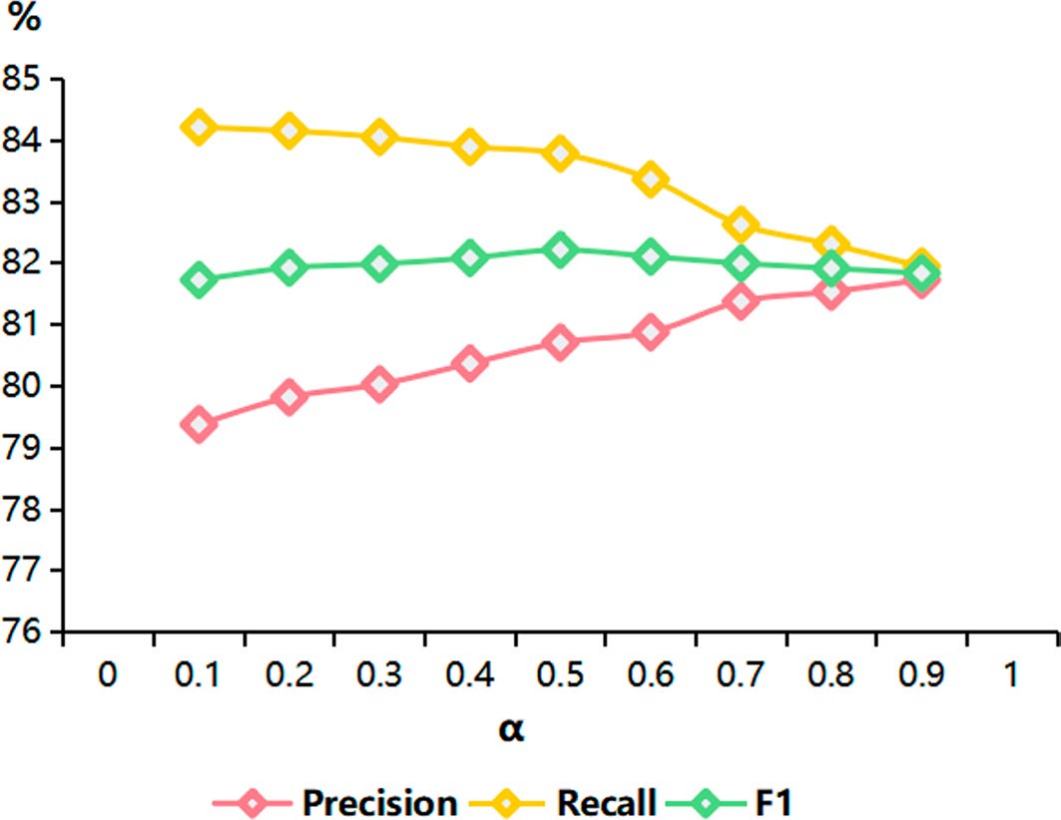
Performance results of the BioLSL model according to the hyperparameter $\alpha$

We conduct an experiment to evaluate the hyperparameter $\alpha$ given in Equation (7) based on the MLEE dataset. Figure 6 shows the performance results of the proposed BioLSL model according to the various values of $\alpha$. In BioLSL, $\alpha$ controls the contributions from Label-Context Aware Representation (LCAR) and Label-Trigger Aware Representation (LTAR) for the combined semantic representation. In particular, we can observe that as $\alpha$ increases from 0.1 to 0.9, the precision of BioLSL also increases due to an increased contribution from LCAR, while the recall decreases due to a reduced contribution from LTAR. Based on the experimental results, we set $\alpha = 0.5$ as it achieves the best F1 performance. It reflects a balanced contribution from both semantic representations for biomedical event trigger detection.

**Table 5:** A case study based on three sentences taken from the test set of the MLEE dataset

Sentence (1):	Combination studies and vascular endothelial growth factor (vegf) ***secretion*** analyses were performed	
**CRF-PubMedBERT:**	None ✗	
**DMBERT-PubMed:**	None ✗	
**BioLSL (Ours):**	{secretion/Localization} $\checkmark$	
Sentence (2):	We report that neuronal death resulting from focal cerebral ischaemia is significantly ***inhibited*** in rats ***injected*** with a ill receptor antagonist.	
**CRF-PubMedBERT:**	{inhibited/Neg-Regulation} $\checkmark$	{injected/Binding} ✗
**DMBERT-PubMed:**	{inhibited/Neg-Regulation} $\checkmark$	{injected/Binding} ✗
**BioLSL (Ours):**	{inhibited/Neg-Regulation} $\checkmark$	{injected/Planned-Process} $\checkmark$
Sentence (3):	These data confirm the importance of tissue architecture and polarity in malignant ***progression***.	
**CRF-PubMedBERT:**	{progression/Development} $\checkmark$	
**DMBERT-PubMed:**	{malignant/Development} ✗	
**BioLSL (Ours):**	{progression/Development} $\checkmark$	

The ground-truth trigger words are highlighted in bold italic. {X/Y} indicates the predicted trigger word X and the corresponding classified event type Y

### Case study

Table 5 shows a case study for the BioLSL, CRF-PubMedBERT and DMBERT-PubMed models based on three sentences taken from the test set of the MLEE dataset. These models are used for comparison as they employ the same PubMedBERT as the encoder. In sentence (1), the word “*secretion*” serves as a trigger for a Localization event. This trigger is overlooked by both CRF-PubMedBERT and DMBERT-PubMed, probably due to the polysemous nature of “*secretion*” and its unclear role in the sentence. However, with the semantic representations that are able to capture both label-trigger and label-context relationships, the BioLSL model can identify “*secretion*” correctly as the trigger word for the Localization event. Sentence (2) is a more complicated sentence with multiple events. Both CRF-PubMedBERT and DMBERT-PubMed have misclassified “*injected*” as the trigger word for a Binding event in this sentence. In fact, the ambiguous word “*injected*” will trigger a Planned-Process event, which is classified correctly by the BioLSL model. In sentence (3) where “*progression*” will trigger a Development event, DMBERT-PubMed incorrectly identifies “*malignant*” as the trigger word for the Development event, whereas both BioLSL and CRF-PubMedBERT can predict the trigger word correctly. As illustrated from the case study, with label-based synergistic representation learning, our proposed BioLSL model is able to detect the trigger words and classify the event types effectively.

## Error analysis

To highlight the challenges in biomedical event trigger detection and suggest areas for future improvement, we have conducted an error analysis of the BioLSL model based on the MLEE dataset. The errors are classified into the following six types:

- Domain Knowledge Requirements—Some errors arise from the model’s difficulty in leveraging domain-specific knowledge. For example, in the sentence “whereas homogeneous and intense immunoreactivity were observed in large and intermediate size blood vessels, heterogeneity of expression was found in capillaries”, the word “expression” refers to a Gene Expression event. The BioLSL model sometimes fails to infer such specific biomedical contexts without explicit domain knowledge, leading to incorrect or missed event triggers.
- Abberiviations and Short Forms—The BioLSL model occasionally fails to expand or interpret abbreviations and short forms correctly. For instance, “peric dysfunction” where “peric” is short for pericyte, or “SOC activation” referring to “Store-Operated Calcium Channel activation”, can lead to errors. The model needs vocabulary enhancement to handle these domain-specific shorthand notations more effectively.
- Inaccurate Boundaries—Errors can also occur due to inaccurate boundary detection of event triggers. For example, the BioLSL model may detect “cell interaction” instead of the correct “cell-cell interaction”, leading to imprecise event classification.
- Annotation Problems—Problematic annotations in the datasets, such as ambiguous definitions, can lead to some errors. For instance, the term “cytoskeletal collapse” refers to the disintegration or disruption of the cytoskeleton, which can lead to changes in cell shape, motility, and function. While cytoskeletal collapse can contribute to cell death processes, it is specifically the breakdown of the cytoskeletal structure itself and not directly synonymous with cell death. However, it is annotated as a Death event trigger rather than a Breakdown event trigger. This ambiguity in annotation guidelines or inconsistencies in the dataset can misguide the BioLSL model, leading to incorrect event trigger prediction.
- Argument Information Understanding—The BioLSL model sometimes struggles with understanding the argument information required for correct event classification. For example, in the sentence “changes in endothelial cell shape accompanied SOC activation”, the word “change” should be recognized as a Development event trigger rather than a Regulation event trigger. Better semantic understanding and context interpretation are needed for the model to make accurate predictions in such cases.
- Overfitting—The BioLSL model occasionally overfits to specific training examples, leading to misclassifications in lexically similar but contextually different instances. For example, in the sentence “mast cells were found to be unique among the peritoneal leukocytes by virtue of their capacity to enhance profoundly the proliferation of a variety of tumors in vitro”, the word “proliferation” should be identified as a Growth event trigger, but is misclassified as Cell Proliferation. This indicates a need for better generalization in the model leveraging the type labels to accurately handle diverse contexts.

Overall, eliminating these issues requires enhancing the BioLSL model’s ability to incorporate domain knowledge, correctly interpret abbreviations, accurately determine event boundaries, resolve annotation ambiguities, understand argument information, consider event co-occurrence, and avoid overfitting. Future work will focus on these aspects to improve the robustness and accuracy of the BioLSL model in detecting biomedical events.

## Conclusion

In this paper, we propose a novel approach to biomedical event trigger detection, which has achieved state-of-the-art performance without relying on external resources that may not always be available in practice. More specifically, we propose the Biomedical Label-based Synergistic representation Learning (BioLSL) model, which effectively uses pre-defined event type labels by learning their correlations with trigger words and capturing their dependencies on the contextual content for biomedical event trigger detection. Experimental results on three benchmark datasets have demonstrated that our proposed BioLSL model significantly outperforms the current state-of-the-art models, and does so without using additional resources or external linguistic tools. We also show that our approach has an advantage in the data-scarce scenarios, with robust performance even on rare event types with a few examples. This is possibly due to the semantic enhancement with our proposed label-based synergistic mechanism. For further work, we plan to address more challenging problems such as few-shot learning in biomedical event extraction.

## Data Availability

The MLEE dataset used in our experiments is available at https://nactem.ac.uk/MLEE/. The GE09 dataset is available at http://www.geniaproject.org/shared-tasks/bionlp-shared-task-2009. The GE11 dataset is available at https://2011.bionlp-st.org/.

## Footnotes

1 https://chat.openai.com/.
